# Fractionation and Mobility of Thallium in Volcanic Ashes after Eruption of Eyjafjallajökull (2010) in Iceland

**DOI:** 10.1007/s00128-016-1831-6

**Published:** 2016-05-21

**Authors:** Bozena Karbowska, Wlodzimierz Zembrzuski

**Affiliations:** Institute of Chemistry and Technical Electrochemistry, Poznan University of Technology, ul.Berdychowo 4, 61-138 Poznan, Poland

**Keywords:** FIA-DPASV, Mobility, Sequential extraction BCR, Tl, Toxicity, Volcanic ash

## Abstract

Volcanic ash contains thallium (Tl), which is highly toxic to the biosphere. The aim of this study was to determine the Tl concentration in fractions of volcanic ash samples originating from the Eyjafjallajökull volcano. A sequential extraction scheme allowed for a study of element migration in the environment. Differential pulse anodic stripping voltammetry using a flow measuring system was selected as the analytical method to determine Tl content. The highest average content of Tl in volcanic ash was determined in the fraction entrapped in the aluminosilicate matrix (0.329 µg g^−1^), followed by the oxidizable fraction (0.173 µg g^−1^). The lowest content of Tl was found in the water soluble fraction (0.001 µg g^−1^); however, this fraction is important due to the fact that Tl redistribution among all the fractions occurs through the aqueous phase.

Volcanic eruptions are one of the main sources of heavy metals in the environment. The spreading of volcanic ash over a large area is a major problem, which leads to a number of health-related risks to humans, animals and plants. Deposition of volcanic dust on the surface of plant leaves and migration of ash with rain water into the soil may lead to the poisoning of the environment and is a direct threat to livestock (Dawson et al. 2010).

The eruption of Eyjafjallajökull volcano was one of the largest in Europe in recent years. The effects of the eruption were recorded in various places in Europe up to the present day. The crater was inactive during the last 187 years. However, on March 20, 2010, the lava began to flow and there were several series of small eruptions which ejected volcanic ash into the atmosphere. After a brief pause the eruption began again on April 14, 2010, with much more intensity, spreading volcanic ash into the atmosphere up to a height of 9 km. Volcanic ash formed during the volcanic eruption, which consisted of small particles of stone powder with a diameter less than 2 mm, resulting in direct damage to the local ecosystem, but also causing serious disruptions in air traffic. It was proven that the westerly winds spread volcanic ash up to a distance of 3000 km from Iceland (Dawson et al. 2010; Gao et al. 2011). The ash began to fall in the south-eastern part of Iceland on April 14. The spread reached Norway and the United Kingdom on the following day (Gíslason and Alfredsson 2010; Wunderman et al. 2011; Gislason et al. 2011).

Several studies showed that the layer of dust deposited on the soil mainly included aluminum, silicon and oxygen. Compounds such as silicon dioxide and silica can cause disease in miners and stonemasons, which is called silicosis. Other components, which were present at notably excessive concentrations, included magnesium, fluoride, and heavy metals such as iron, nickel, chromium, cadmium and mercury (Gíslason and Alfredsson 2010; Maynard et al. 2010).

Although there is little information regarding this subject, part of the volcanic ash also contained thallium (Tl). Thallium is a soft, malleable metal, with a blue-white colored surface similar in appearance to lead. Due to the similarity of alkali metals, it can replace potassium in biological systems. As a result, Tl can interfere with cellular metabolism by reducing the activity of important enzymes and coenzymes. Furthermore, it is characterized by high toxicity to aquatic organisms, humans and other animals (Dmowski and Badurek 2001; Dmowski et al. 2002; Krasnodębska-Ostręga et al. 2005). Thallium (I) compounds with high solubility in water are readily absorbed through the skin. Thallium is considered as probably carcinogenic to humans (Emsley 2006). It is highly toxic to the biosphere, with toxicity higher than that of mercury, cadmium, lead and copper (Peter and Viraraghavan 2005). Moreover, it exhibits mutagenic, carcinogenic and teratogenic properties. Stomach and intestinal ulcers, alopecia, and polyneuropathy are considered classic syndromes of Tl poisoning (Xiao et al. 2007). Ingestion of more than 1.5 mg per kg of body weight (approximately 0.2–1 g) can be fatal. Thallium salts are rapidly and almost completely absorbed through the gastrointestinal route. No symptoms are evident, at least at the beginning of intoxication, thereby preventing accurate diagnosis of early Tl poisoning, and thus the application of appropriate therapeutic measures (Lech and Sadlik 2007). Skin contact, ingestion, and introduction into the respiratory tract are very dangerous and cause irritation of the gastrointestinal tract and nervous system disorders. Thallium poisoning can cause changes in blood chemistry, liver, kidney, intestine and testis tissue. Additionally, it results in abdominal pain, diarrhea and tingling of the extremities. Due to the notable element of risk, the content of thallium is systematically measured in environmental samples (e.g. river water or groundwater) in order to prevent its uncontrolled spread (Lukaszewski et al. 2010; Wojtkowiak et al. 2016).

Mobility, toxicity and bioavailability of metals depends mainly on the chemical form in which they occur, and how they bind to the soil matrix. Metals in uncontaminated soils mainly form silicates and primary minerals, resulting in a relatively immobile form (Yang et al. 2005). In soils already contaminated with metals additional metal ions are more mobile, due to their weaker binding to other components of the soil (Lukaszewski et al. 2003). Determination of total metal content in samples provides information which allows for an assessment of the degree of soil contamination associated with the presence of metals. The total concentration of Tl in selected elements of the environment is only an initial measure for the estimation of health hazards associated the presence of this compound. However, only the study of distribution of metals in the appropriate fractions allows for the determination of possible effects on the environment (Vaněk et al. 2011; Vaněk et al. 2010a, b). It is important to establish the mobility of Tl. Such data may be obtained by utilization of sequential extraction. Previous reports indicated that the major fraction of Tl was entrapped in the aluminosilicate parent matter (Lukaszewski et al. 2012) and became immobile in the environment. Therefore, it was hypothesized that a similar effect would be observed in case of volcanic ash originating from Eyjafjallajökull.

The aim of this study was to determine the Tl concentration in fractions of volcanic ash samples originating from the Eyjafjallajökull volcano. The number of reports dedicated to the determination of Tl in the environment is limited and the scale of associated health hazards is poorly understood. To the best of our knowledge this is the first study to characterize the fractions of Tl present in soil contaminated with volcanic ash.

## Materials and Methods

The following apparatuses were used during the experiments: an ISM596D peristaltic pump (Ismatec, Jona, CH) with a flow rate of 2 mL/min, a PalmSens3 μ-AUTOLAB ECOCHEMIE electrochemical analyzer (PalmSens, Utrecht, NL), a mercury film electrode based on epoxy resin impregnated graphite (which was used as a working electrode), a saturated calomel electrode (which was the reference electrode) and a platinum wire (which was the auxiliary electrode). Other laboratory equipment used during all measuring steps were: a pH meter, a magnetic stirrer, and a technical balance.

The solution for the electrolytic production of film consisted of 0.1 M potassium nitrate and 0.05 mM Tl (I) nitrate. A starting solution of 0.2 M EDTA was used to obtain an alkaline electrolyte solution of 0.05 M EDTA and 0.1 M ascorbic acid. Acetic acid (0.1 M) was used in sequential extraction of soil samples for the determination of Tl in the carbonate fraction. Ammonium hydroxide (0.1 M) was used for the determination of Tl compounds in the reducible fraction. A 1 M ammonium acetate solution was used for the digestion of soil samples, during the determination of Tl. Hydrofluoric acid (73 %) was used to dissolve the sample and pre-stripping of volatile compounds. Afterwards the sample was treated with 65 % nitric acid (V) and 30 % hydrogen peroxide. All chemical reactants were obtained from Sigma Aldrich (Poznan, PL). GBW 07401 Chinese soil, certified for total content of Tl (1.0 ± 2.0 µg × g^−1^) was used as reference material (Chinese National Standard Reference Materials, Beijing, CN).

The research material was derived from volcanic ash originating from the volcano Eyjafjallajökull in Iceland. Four samples (A, B, C and D) were analyzed with three replicates per sample (a total of 12 measurements). The first part of samples was subjected to determination the total content of Tl in volcanic ash (the water soluble, acid soluble/exchangeable, reducible and oxidized fractions). The second part of samples, was mineralized before the process of sequential extraction of Tl in volcanic ashes (the fraction entrapped in parent matter). Prior to the determination, the samples the volcanic ash were subjected to a granulometric analysis with the use of set of pre-defined sieves. The results of the analysis are presented in Table 1.

**Table 1:** Granulometric analysis of the studied volcanic ash samples

Fraction size (mm)	Ratio (%)
0.5–2	0.1
0.25–0.5	16.7
0.1–0.25	29.5
0.05–0.1	39.6
0.02–0.05	13.2
<0.02	0.9

**Fig. 1:**
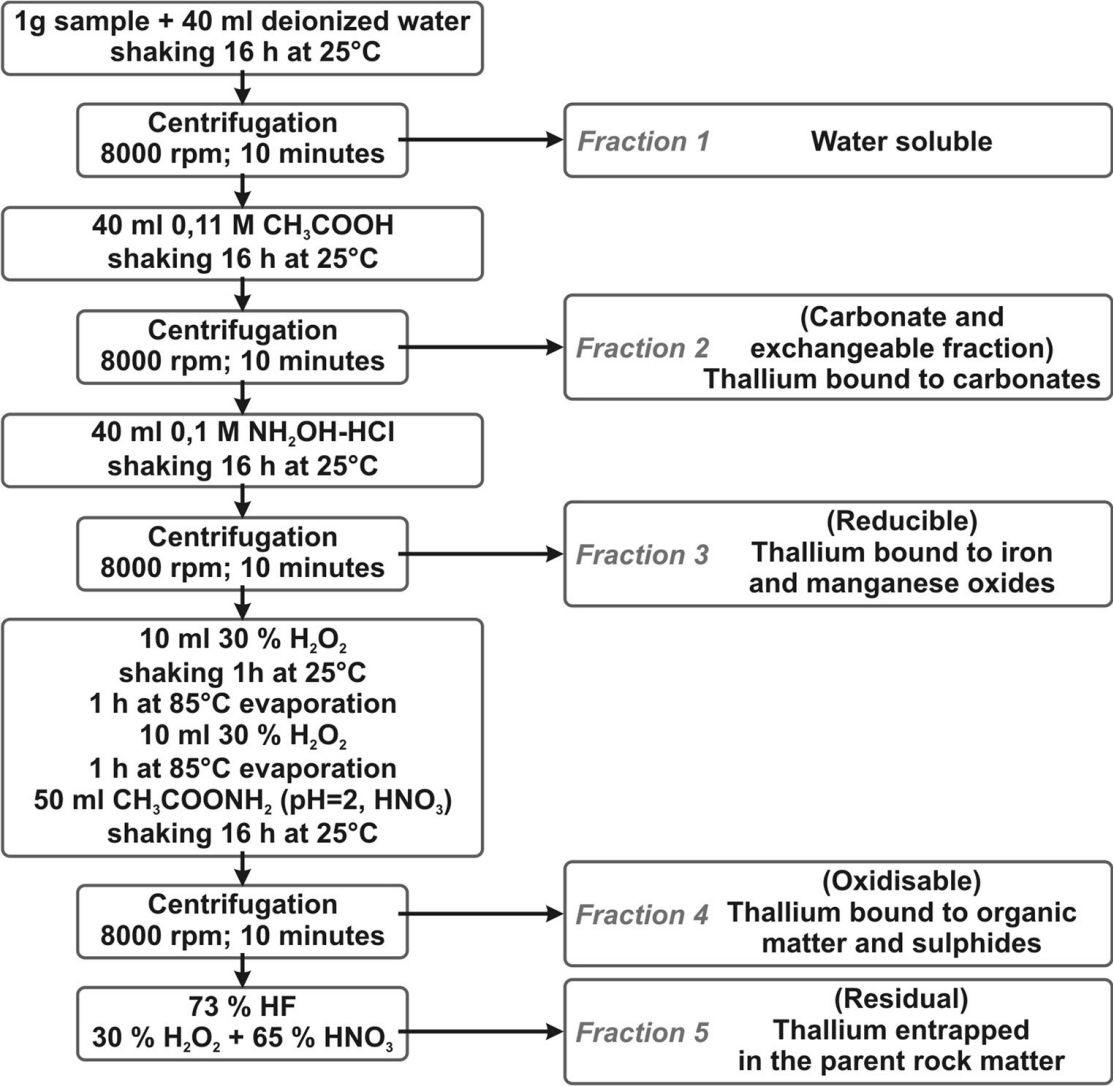
Community Bureau of Reference (BCR) extraction scheme

The use of Community Bureau of Reference (BCR) extraction scheme (Fig. 1) was employed as the analytical method for determination of Tl in subsequent fractions (Vaněk et al. 2010a, b). The water soluble fraction was obtained by treating 0.25 g of volcanic ash sample with 10 mL of demineralized water in a 100 mL conical flask and shaken at 25°C for 16 h. The mixture was centrifuged and the solution was transferred to a 25 mL volumetric flask. Afterwards, 6.25 mL of 0.2 M EDTA was added and the flask supplemented with water to the mark.

The acid soluble/exchangeable fraction was obtained by treating the residual sample from the previous step with 10 mL of 0.11 M acetic acid in a 100 mL conical flask and shaken at 25°C for 16 h. The mixture was then centrifuged and the solution transferred to a 25 mL volumetric flask. The sediment sample was treated with 2.5 mL of water and shaken for 10 min, then centrifuged and the solution was added to the solution of the fraction. The pH of the solution was adjusted to 4.5 % with 25 % aqueous ammonia solution or 2 M nitric acid. Afterwards 6.25 mL of 0.2 M EDTA was added and the flask supplemented with water to the mark.

The reducible fraction was obtained by treating the residual sample from the previous step with 10 mL of 0.1 M hydroxylamine hydrochloride, followed by adjusting the pH to 2 with 2 M nitric acid in a 100 mL conical flask and shaking at 25°C for 16 h. The mixture was centrifuged and the solution was transferred to a 25 mL volumetric flask. The sediment sample was treated with 2.5 mL of water, shaken for 10 min, and then centrifuged. The solution was added to the solution of the fraction. Afterwards, 2.5 mL of 1 M ascorbic acid was added to the joint solutions. The pH was adjusted to 4.5 with 25 % aqueous ammonia solution or 2 M nitric acid, then 6.25 mL of 0.2 M EDTA was added and the flask filled with water.

The oxidized fraction was obtained by treating the residual sample from the previous step with 2.5 mL of 30 % hydrogen peroxide in a 100 mL conical flask, and then shaking at 25°C for 1 h and at 85°C for 1 h. The mixture was evaporated to the volume of 1–2 mL. The next 2.5 mL portion of 30 % hydrogen peroxide was added, the mixture shaken at 85°C for 1 h, and the mixture evaporated until dry. The residue was treated with 12.5 mL of 1 M ammonium acetate and the pH value was adjusted to 2 with 2 M nitric acid, and then shaken at 25°C for 16 h. The mixture was centrifuged and the solution transferred into a 25 mL volumetric flask. The sediment sample was treated with 2.5 mL of water, shaken for 10 min and centrifuged. The solution was added to the solution of the fraction. Afterwards 2.5 mL of 1 M ascorbic acid was added to the joint solutions. The pH was adjusted to 4.5 with 25 % aqueous ammonia solution or 2 M nitric acid, followed by the addition of 6.25 mL of 0.2 M EDTA. Water was then added to fill the flask to its mark.

The fraction entrapped in parent matter was obtained by placing a dried 0.25 g sample of volcanic ash into a Teflon beaker, and digesting it by adding 2 mL of 73 % hydrofluoric acid. After 2 h, 0.6 mL of hydrofluoric acid were added. The solution was heated until evaporation, then 1 ml of 67 % nitric acid and 2.5 mL of 30 % hydrogen peroxide in portions of 0.5 mL were added. After evaporation of the solution, another portion of 1 mL of nitric acid was added. Afterwards, it was covered with a watch glass and heated for 3 h. The soil solution was then filtered. The filtrate was mixed with 2.5 mL of 1 M ascorbic acid and 6.25 mL of 0.2 M EDTA. Then, after adjusting the pH value to 4.5 using ammonium solution, the solution was transferred to a 25 mL flask and supplemented with water.

**Fig. 2:**
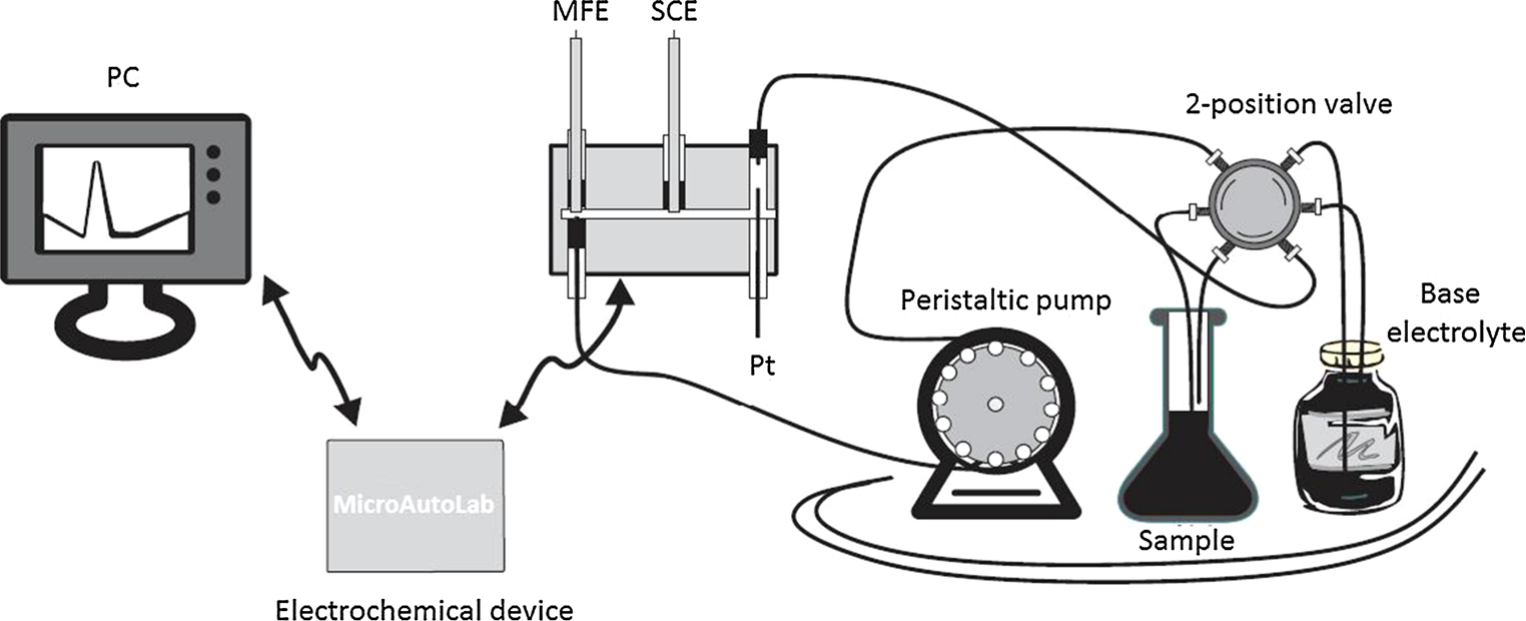
Flow-injection voltammetric system. *PC* personal computer, *MFE* mercury film electrode, *SCE* saturated calomel electrode

The obtained solution was used to determine the total content of Tl in the volcanic ash using flow injection analysis – differential pulse anodic stripping voltammetry (FIA-DPASV) (Lukaszewski et al. 2010). Determination of Tl by FIA-DPASV was carried out according to the following protocol (Fig. 2). Peristaltic pump with forced flow was used with constant flow rate of 2 mL/min. The sample was aspirated from the 10 mL sample vial into the measuring vial, which contained three electodes: a mercury film electrode based on epoxy resin impregnated graphite was used as a working electrode, a saturated calomel electrode was the reference electrode and platinum wire was the auxiliary electrode (Lukaszewski and Zembrzuski 1992, 2010).

The use of a flow measuring system made it possible to avoid the problem of depletion of the sample solution caused by the limited capacity of the measuring vessel and allowed for the circulation of the electrolyte. A 0.05 M EDTA solution was used as the base electrolyte.

The operating parameters (i.e., concentration, potential and time) for the measurements were selected based on a series of previous studies (Jakubowska et al. 2008).

Determination of peak height, which depended on the potential of Tl concentration, allowed for the selection of -0.9 V as the optimum potential for the study. The dependence between the peak concentration of Tl and time allowed for determination of time necessary for sample concentration to achieve a readily measurable amount in the subsequent analysis. The resulting linear dependence of peak height on its concentration of Tl showed no signs of depletion of Tl.

After the mineralization procedure, the concentration of Tl in the samples (A, B, C, and D) was estimated on the basis of several standard additions (typically three additions). All measurements were carried out in triplicate (three independent measurements for samples A, B, C, and D), which were used to calculate standard deviation values (based on a total of 12 measurements).

In order to control the analytical quality, the precision of the method was determined with a reference material – soil GBW 07401. Nine independent trials were conducted for the reference material in order to determine the Tl content. The average content of Tl was at 0.90 ± 0.14 µg/g (with a minimum of 0.76 and a maximum of 1.1 µg/g). The recovery of Tl was at 90 %.

## Results and Discussion

Volcanic ash sediments were processed in accordance with the modified BCR procedure. The results are shown in Table 2. Additionally, total Tl concentration was determined independently in the investigated samples.

**Table 2:** Tl concentration (µg g^−1^) in volcanic ash fractions (obtained by sequential extraction and by total independent measurement

Ash sample	Fraction					Total	Total independent measurment
	Water soluble	Acid soluble/exchangeable	Reducible	Oxidizable	Entrapped in parent matter		
A	0.0008	0.004	0.01	0.201	0.359	0.575	0.499
B	0.0007	0.005	0.02	0.159	0.365	0.550	0.520
C	0.0016	0.004	0.01	0.146	0.257	0.419	0.441
D	0.0016	0.004	0.01	0.187	0.335	0.538	0.442
Blank	0.00008	0.0001	0.0008	0.0001	0.0003		0.0014
Average	0.0012	0.004	0.0125	0.173	0.329	0.520	0.471
SD	0.0004	0.001	0.005	0.025	0.049		0.046
Percent of Total Tl	0.23	0.77	2.4	33.27	63.27		

**Fig. 3:**
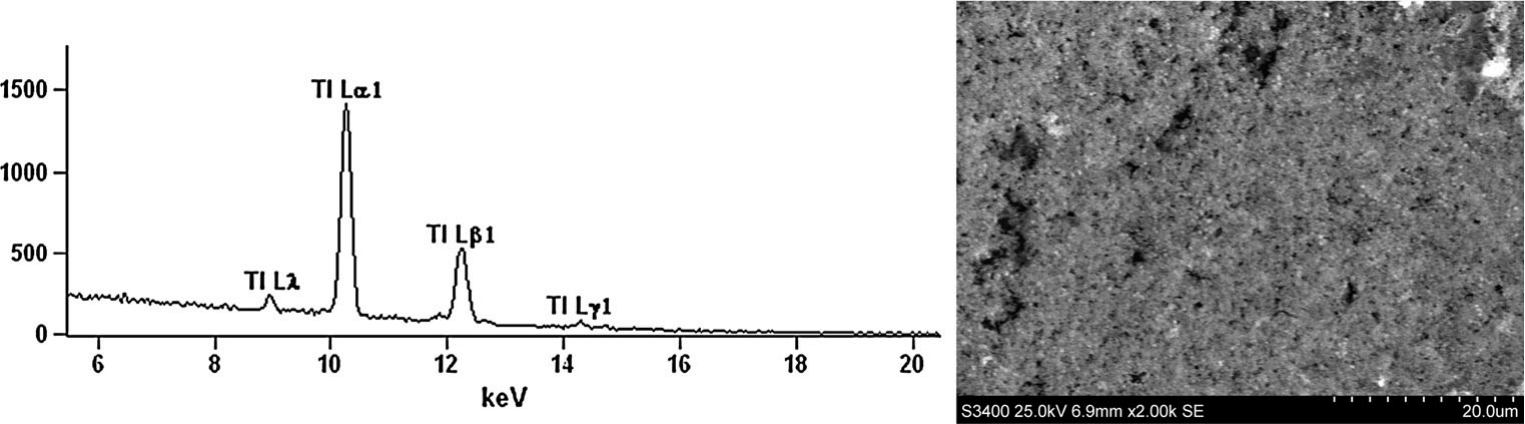
Energy dispersive spectra (*left*) and scanning electron micrograph of electrode surface (*right*)

The results shown in Fig. 3 indicate that Tl formed a deposit on the electrode surface. The conclusion based on the performed investigation was that the majority of Tl in the investigated volcanic ash sediment samples (63 %) was entrapped in the alumosilicate parent matter; i.e., it was entirely unavailable (Table 1). Only total destruction of this residual fraction with hydrofluoric acid makes this Tl available. This conclusion strongly supported the hypothesis that Tl would be mainly entrapped in residual parent matter. This is a significant observation; the potential risk of the toxic effect of Tl is strongly limited due to low mobility. The mobile Tl i.e. Tl contained in four basic ally soluble sediment fractions comprised only 36 % of the total content. The order for concentrations of Il in the four mobile sediment fractions was: water soluble fraction < exchangeable fraction < reducible fraction < oxidizable fraction. It is worth emphasizing that the combined Tl concentration in the most mobile water soluble and acid soluble/exchangeable fractions was very small and varied between 0.23 % and 0.77 % of the total Tl concentration in the investigated samples.

The water soluble fraction varies from 0.0007 to 0.0016 µg g^−1^. Determination of the water fraction is important due to the fact that Tl redistribution among all the fractions occurs through the aqueous phase (Karbowska et al. 2014).

The acid soluble/exchange able fraction varied from 0.004 to 0.005 µg g^−1^. The fraction of ion exchange/carbonate dissolution indicates an acidic environment. The exchangeable fraction includes related metals in soil solution and the associated constant fraction of the soil on the basis of physical and chemical adsorption and ion exchange sorption. This fraction mainly includes metals that are retained on the soil surface by relatively weak electrostatic forces, and metals that may be released during ion exchange. It usually accounts for about 2 % of the total content of elements present in the soil. The carbonate fraction includes heavy metal carbonates and forms adsorbed with calcium carbonate, sulphates and phosphates (Filgueiras et al. 2002). This fraction is very sensitive to changes of pH, which is why it is important that this stage of extraction was carried out at pH5 (Kuokkanena et al. 2006).

The reducible fraction varies from 0.01 to 0.02 µg g^−1^. It describes the fraction of reducible metal oxides bound with iron and manganese hydroxides. The fraction associated with oxides of iron and manganese includes metals absorbed to hydrated oxides of iron and manganese and in the form of thin layers surrounding minerals (this fraction is unstable with a deficit of oxygen or a change in redox potential). Iron and manganese oxides can strongly bind metals. They are also a component of the sorption complex. The most effective reagents that are likely to capture the total metal content associated with iron and manganese oxides contain both a reducing reagent and a ligand capable of maintaining the released ions in dissolved form. The effectiveness of the reagent depends on its reduction potential (Jakubowska et al. 2007). Crowding consists of one or several stages, involving the separation of amorphous or crystalline forms of iron and manganese oxides (Lukaszewski and Zembrzuski 1992).

Oxidizable fraction varies from 0.146 to 0.201 µg × g^−1^. The oxidizable fraction provides information regarding the metal associated with organic compounds and sulfides, which is the form of Tl released into the environment under oxidative conditions (Lukaszewski and Zembrzuski 1992). This fraction includes metals associated with various forms of organic matter, mainly humic and fulvic acids and sulfides. The elements may interact with soil organic matter, (i.e. humic substances) and become entrapped in the soil matrix, depending on the environmental conditions (e.g. the pH) (Varrault and Bermond 2002).

The fraction of Tl entrapped in the parent mater varies from 0.257 to 0.365 µg g^−1^. Determination of Tl content in the residual fraction is important in evaluating the overall balance of Tl in all fractions. This fraction was compared with the summary concentration of Tl. Determination of the residual fraction was obtained after treatment of the soil sample with strong acid, which ensures accurate results according to the results of previous studies (Lukaszewski et al. 2010).

The total concentration of Tl determined in the volcanic ashes (0.4–0.52 µg g^−1^) was two times higher compared to that in Tamar estuarine sediments in England (0.08–0.22 µg g^−1^) (Anagboso et al. 2013). The established concentrations range was similar to that reported for soil floodplain terraces in Poland (0.38–0.44 µg g^−1^) (Jakubowska et al. 2007), tsunami sediments in Thailand (0.38–1.08 µg × g^−1^) (Lukaszewski et al. 2012), soils in the Czech Republic (0.43–0.79 µg g^−1^) (Vaněk et al. 2010a, b), as well as soils derived from magmatic rocks (0.32–1.69 µg g^−1^), metamorphic rocks (0.26–1.0 µg g^−1^), plastic rocks (0.04–0.88 µg g^−1^) and calcareous rocks (0.11–21.6 µg g^−1^) in France (Tremel et al. 1997).

The Community Bureau of Reference (BCR) procedure (Vaněk et al. 2010a, b) allowed for an evaluation of how the soil metal might behave under the influence of changing environmental conditions. Depending on their forms metals are more or less mobile and thus bioavailable. Assessment of the behavior of heavy metals in soils should no longer be based only on total heavy metal content – diagnosis and characterization of various forms/fractions of a given element is also necessary. Most of the standards for the maximum concentrations of heavy metals in both soils and water have continued to focus only on their total content. However, such data are insufficient for assessment of their activity and toxicity (Jeske and Gworek 2011).

The majority of Tl in the analysed volcanic ash (63 %) was entrapped in the alumosilicate parent matter, where it is entirely immobile. Only the total destruction of this residual fraction with hydrofluoric acid made this Tl available. This conclusion strongly supported the hypothesis that generally Tl would be mainly entrapped in an alumosilicate parent matter. Studies regarding the characterization of Eyjafjallajökull volcanic ash particles revealed the following bulk mineral composition: SiO_2_ (57.9 %), Al_2_O_3_ (14.9 %), FeO (9.8 %), CaO (5.5 %), Na_2_O (5.0 %), MgO (2.3 %), K_2_O (1.8 %), TiO_2_ (1.8 %), P_2_O_5_ (0.5 %), MnO (0.2 %) (Gislason et al. 2011). The high content of SiO_2_ and Al_2_O_3_ corresponds well with the findings regarding the entrapment of Tl in alumosilicate parent matter. Furthermore, the 2 % content of K_2_O may be associated with the increased content of Tl in the tested samples, since the geochemical behaviour of Tl is analogous to that of K (Kabata-Pendias 2010). Tl exhibits high affinity to various minerals, which may lead to release of K from such minerals and inhibition of its activation energy.

In conclusion, it appears that the majority of the Tl originating from volcanic ash was not readily bioavailable in the environment. However, some plant species (such as white mustard, which is commonly used as livestock fodder) are capable of accumulating notable amounts of Tl-based compounds from soil, even with its limited mobility (Vaněk et al. 2010a, b). This phenomenon may be of importance in terms of environmental contamination, as uptake of Tl by such plants may lead to their direct introduction into the food chain.
